# Supplementary figures and images for: The associations between thermal variety and health: Implications for space heating energy use
Source: PLoS One. 2020 Jul 22;15(7):e0236116. doi: 10.1371/journal.pone.0236116 (PMC7375518; doi:10.1371/journal.pone.0236116)

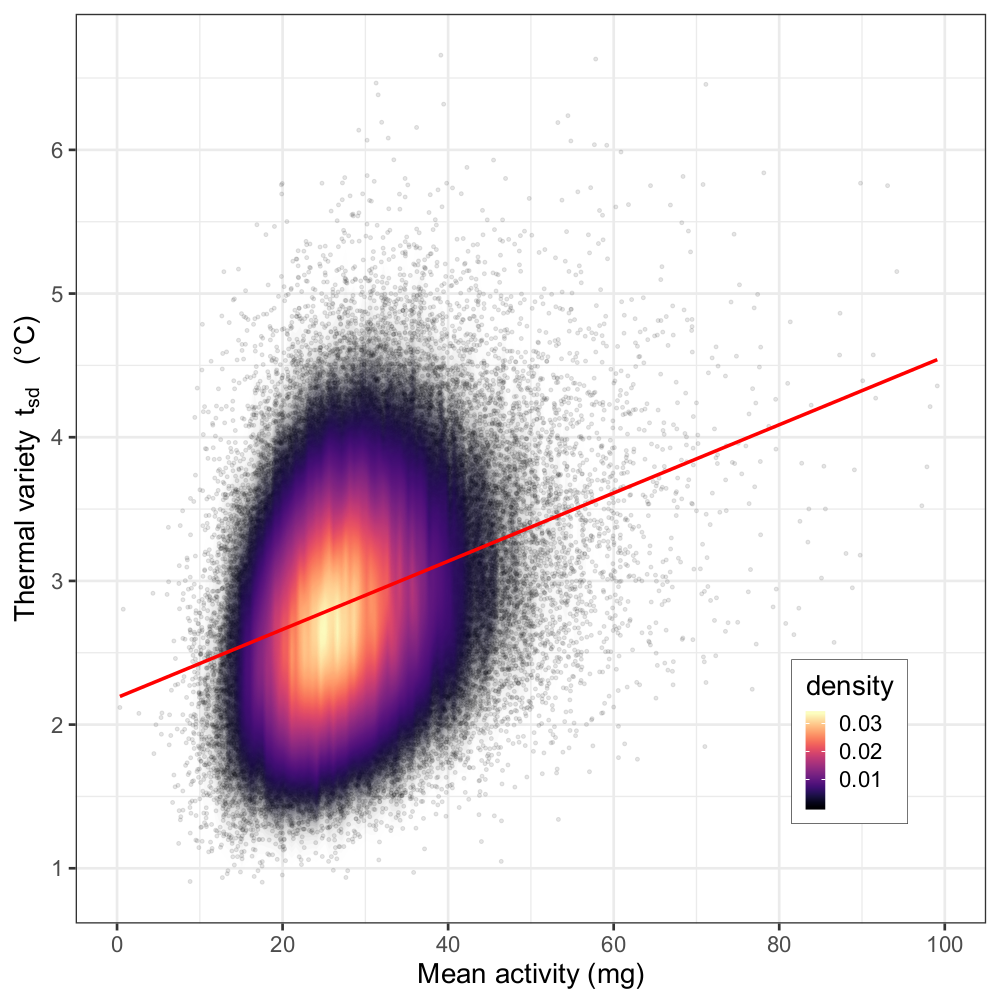

Supplement: S1 Fig — tsd=0.02a¯+2.19, where a¯ is the mean recorded activity for the study week. (TIF) [file pone.0236116.s001.tif]

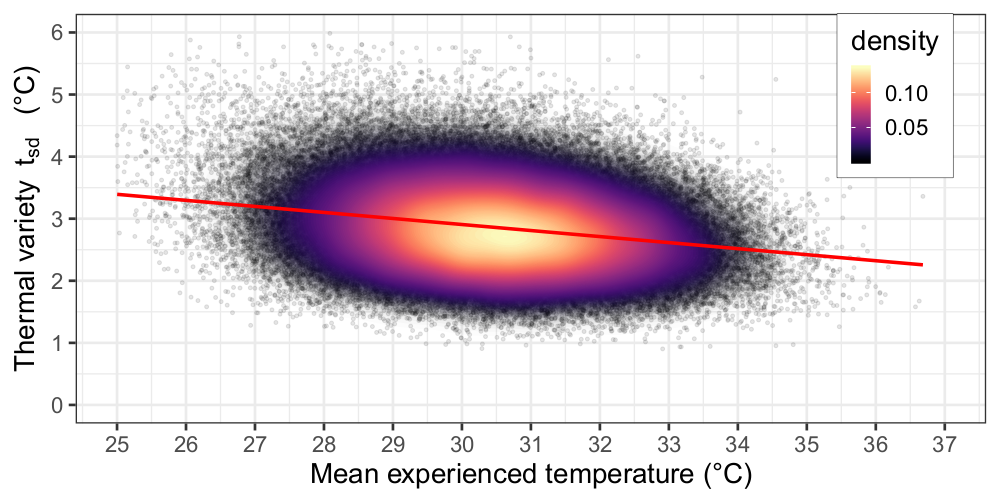

Supplement: S2 Fig — tsd=−0.1t¯+5.8, where t¯ is the mean experienced temperature. (TIF) [file pone.0236116.s002.tif]
