# Supplementary material for: The associations between thermal variety and health: Implications for space heating energy use
Source: PLoS One. 2020 Jul 22;15(7):e0236116. doi: 10.1371/journal.pone.0236116 (PMC7375518; doi:10.1371/journal.pone.0236116)
Supplement: Attachment — Submitted filename: Reviewer Comment_SerebeGebrie.docx [file pone.0236116.s003.docx]

**Abstract** - No comment.

**Introduction**

**(Page 4, L61-64**) – It shows thermal asymmetries and local air movement would offer opportunities for personal control. Would you explain a little about how temperature is controlled or regulated in the UK? what are the major problems with the current system?

**Methods**

In this study, you have used two models. What is the rationale to use these two models? It seems model 2 is more reliable than model 1. Self-reported health satisfaction could be more subjective and has a higher risk of bias. It would be clear if you could add the reason why you have used these two models.

In this paper, there are some abbreviations and subject-specific words. Eg. ICD, HOBO, UCL’s cluster. I suggest using abbreviations and operational definitions. Otherwise, you may define or write in full text when first use.

**(Page 5, L91&92) –** “While the data were being processed, 4 participants withdrew from the study, and their data were deleted.”. This sentence gives me a little confusion. Have you used primary data directly from participants? If so, you need to add ‘Ethical consideration’.

**Result**

The reader may expect to see why household income was not associated with health risks in model 1, but it did in model 2. What could be the reason for this variation? Please include it in your discussion.

**Discussion** – No comment

**Reference** – No comment
